# Computational Site Saturation Mutagenesis of Canonical and Non-Canonical Amino Acids to Probe Protein-Peptide Interactions

**DOI:** 10.3389/fmolb.2022.848689

**Published:** 2022-04-14

**Authors:** Jeffrey K. Holden, Ryan Pavlovicz, Alberto Gobbi, Yifan Song, Christian N. Cunningham

**Affiliations:** ^1^ Department of Early Discovery Biochemistry, Genentech, South San Francisco, CA, United States; ^2^ Cyrus Biotechnology, Seattle, WA, United States; ^3^ Department of Discovery Chemistry, Genentech, South San Francisco, CA, United States

**Keywords:** noncanonical, peptide, macrocycle, design, rosetta

## Abstract

Technologies for discovering peptides as potential therapeutics have rapidly advanced in recent years with significant interest from both academic and pharmaceutical labs. These advancements in turn drive the need for new computational tools to design peptides for purposes of advancing lead molecules into the clinic. Here we report the development and application of a new automated tool, AutoRotLib, for parameterizing a diverse set of non-canonical amino acids (NCAAs), N-methyl, or peptoid residues for use with the computational design program Rosetta. In addition, we developed a protocol for designing thioether-cyclized macrocycles within Rosetta, due to their common application in mRNA display using the RaPID platform. To evaluate the utility of these new computational tools, we screened a library of canonical and NCAAs on both a linear peptide and a thioether macrocycle, allowing us to quickly identify mutations that affect peptide binding and subsequently measure our results against previously published data. We anticipate *in silico* screening of peptides against a diverse chemical space will be a fundamental component for peptide design and optimization, as more amino acids can be explored in a single *in silico* screen than an *in vitro* screen. As such, these tools will enable maturation of peptide affinity for protein targets of interest and optimization of peptide pharmacokinetics for therapeutic applications.

## Introduction

Peptides are an emerging therapeutic modality composed of 4–100 amino acids (Muttenthaler et al., 2021) that are currently utilized as antifungal, antibacterial, anticancer, and hormone therapies (Fosgerau and Hoffmann, 2015). Many of these peptide-based drugs have been derived from natural products, hormones, or fragments of larger proteins (McGregor, 2008); however, in recent years the scaffolds for discovery have broadened to include stapled helices, macrocycles, and peptide conjugates (Muttenthaler et al., 2021). The discovery of new peptide therapeutics is challenging and often requires screening a large peptide library using traditional approaches like phage or yeast display, both of which are limited by the sequence diversity that can be encoded within the library. With the recent advent of mRNA display combined with random non-standard peptide integrated discovery (RaPID) technology to encode peptides that contain NCAAs (Heinis and Winter, 2015; Maini et al., 2016; Zhu et al., 2017), library diversity has dramatically expanded and enabled discovery on a variety of different peptide scaffolds that contain multiple non-canonical amino acids (NCAAs). Compared to their fully canonical counterparts, NCAA-containing peptides have been shown to increase peptide binding affinity (Zhang et al., 2014), protease resistance (Baumann et al., 2017), and membrane permeability (Walport et al., 2017). Moreover, advances in solid-phase peptide synthesis have expanded the chemical diversity of peptides that can be synthesized. Therefore, our ability to discover and evaluate chemically diverse peptides has radically improved in the past decade. Nonetheless, optimization of each peptide is a time consuming and laborious process that requires multiple cycles of discovery and peptide synthesis. In order to accelerate design and optimization of peptides with NCAAs new computational tools are required to guide library design, affinity maturation, and pharmacokinetic optimization of lead peptide candidates as the chemical space of NCAAs is substantially greater than the chemical space sampled by canonical amino acids (Viarengo-Baker et al., 2021).

Computational design (Schymkowitz et al., 2005; Maguire et al., 2020; Chemical Computing Group, 2022) and simulation (Khoury et al., 2014) software packages have become a valuable tool for protein and peptide engineers to evaluate macromolecular ternary structures. For the software package Rosetta, peptide design has been well benchmarked (Sood and Baker, 2006; Smith and Kortemme, 2010; Hosseinzadeh et al., 2017; Sitthiyotha and Chunsrivirot, 2020; Mulligan et al., 2021), including the use of NCAAs (Renfrew et al., 2012; Drew et al., 2013). *In silico* screening of NCAAs in a linear or cyclic peptide offers multiple advantages: 1) the non-covalent interactions between the peptide and its binding partner can be tuned to improve binding affinity and 2) peptide properties can be predicted and modulated to improve cell permeability and *in vivo* stability of the peptide. Considering that a peptide can be further modified to include D-amino acids, N-methyl amino acids, peptoids, beta amino acids and gamma amino acids (Drew et al., 2013; Adaligil et al., 2021a, 2021b), the chemical space that a peptide with NCAAs can sample is enormous (Otvos and Wade, 2014; Viarengo-Baker et al., 2021). Therefore, robust strategies are needed for parameterizing NCAAs within design algorithms that incorporate these expanded amino acid libraries.

Here we report the development and application of new automated tools for parameterizing exotic amino acids, N-methyl amino acids, and peptoids with ≤4 heavy atom torsion angles for use in the computational design program Rosetta. The development of these tools may also prove useful for computational studies of post-translational modifications and protein engineering strategies with NCAAs. For our purposes, we evaluated the geometries of the generated rotamer libraries using quantum mechanical conformational analysis and tested a set of parameterized NCAAs with a Rosetta design protocol. In order to complete *in silico* studies on peptide scaffolds containing a thioether linkage we also developed additional computational tools for thioether cyclized peptides to be designed within Rosetta. Importantly, these new computational tools enable peptides discovered from large combinatorial libraries, like the RaPID platform, to be screened against a computationally defined NCAA library. To evaluate NCAAs within Rosetta we utilized the NMR structure of the linear peptide PUMA bound to MCL-1 (PDB 2ROC) and the crystal structure of macrocycle CP2 bound to KDM4 (PDB 5LY1) as starting points for *in silico* site saturation mutagenesis studies. Site saturation mutagenesis on the aforementioned peptides required that every NCAA defined within our library was evaluated at each peptide residue position. The results of these computational studies were then compared to previously published experimental site saturation mutagenesis studies on both PUMA and CP2 peptides (Rogers et al., 2018). Moreover, we demonstrate that these computational tools can also be used to evaluate protein-peptide interactions to screen chemical space beyond what is feasible experimentally. We anticipate these tools will be useful for designing optimized structure-based libraries for empirical discovery efforts to improve target engagement and pharmacokinetics of peptides.

## Materials and Methods

### Parameterization of NCAAs

We developed an automated workflow to generate Rosetta parameters for NCAAs with minimal user input. The protocol requires only a SMILES string for the NCAA of interest along with the total charge of the side chain. The NCAA needs to be capped at the N and C termini with an acetyl and N-methyl group, respectively, in order to unambiguously differentiate the backbone from side chain atoms. From there, a three-dimensional structure of the NCAA is built with the OMEGA module of OpenEye (OMEGA 4.1.1.1, 2021) and partial charges are assigned to all atoms according to the AM1BCCELF10 method applied by the QUACPAC module of OpenEye (QUACPAC 2.1.2.1, 2021). Additional corrections are made to maintain backbone charge consistency with the REF2015 score function in Rosetta (Park et al., 2016). Five different sets of backbone charges are used, depending on the general class of NCAA detected by the automated tools: peptoid, N-methyl alpha amino acid, N-cyclized (e.g., piperidinecarboxylic acid), Cα-cyclized (e.g., 1-aminocyclopropane-1-carboxylic acid), Cα-branched (e.g., 2-aminoisobutyric acid), and the standard canonical alpha amino acid backbone. The backbone partial charges for each class of NCAA are listed in Supplementary Table S8. After backbone charge standardization, any charge difference from the total expected charge is equally distributed among the side chain atoms directly connected to the backbone.

After Rosetta atom types are assigned and rotatable side chain bonds are detected by the molfile_to_params_polymer.py script that is part of the Rosetta tools repository, a backbone-dependent side chain rotamer library is then generated. Determination of side chain conformational preferences for each phi/psi combination, sampled at 10° intervals, follows the method used by the MakeRotLib protocol in Rosetta (Renfrew et al., 2012), wherein for a backbone with fixed phi and psi angles, each side chain rotatable bond, **X**_i_, is exhaustively sampled in regular small-degree intervals of 1°, 5°, 15°, and 30° prior to an energy minimization step. The resulting points in **X** space are then clustered around expected idealized torsion values, with the new **X** values for each centroid representing the rotamer conformations used in the rotamer library.

Our method deviates from the previously reported MakeRotLib protocol (Renfrew et al., 2012) by incorporating tools from OpenEye. In the interest of minimal user input, the idealized **X** angles for a side chain are automatically assigned using the values from the original OpenEye OMEGA torsion library (OMEGA 4.1.1.1, 2021). For select cases where the atom types are of specific types, such as a **X**_i_ that rotates a Csp^3^-Csp^3^ bond, the parameterization tool overrides the expected minima of the torsion library. The sampling of side chain conformations on a fixed backbone is performed with OMEGA from OpenEye (OMEGA 4.1.1.1, 2021). The degree of initial side chain sampling is dependent upon the number of rotatable bonds in the side chain. Sampling intervals of 1°, 5°, 15°, and 30° are used depending on whether the side chain has 1, 2, 3, or 4 rotatable bonds respectively, excluding methyl and hydroxyl groups. After generating all possible conformations with the prescribed sampling interval, the conformations are energy minimized with the MMFF94S force field using the SZYBKI module of OpenEye tools (SZYBKI 2.3.0.0, 2021). Specifically, the steepest descent optimizer is used with a gradient tolerance of 0.001 kcal/mol in conjunction with the Sheffield solvent model with a dielectric constant of 3.0. Following the first round of energy minimization, each conformer is assigned to a cluster center that is initially defined by the expected energy minima from the OpenEye torsion library. Boltzmann-weighted centroids are then recalculated for each cluster, using the computed MMFF94S energies and a k_B_T value of 4.0. With the updated cluster centers, the energy-minimized side chain conformations are reclustered based on their measured distance in **X**-space to the new cluster centers as illustrated in Supplementary Figure S1. One final Boltzmann-weighted centroid calculation yields the final set of rotamers for the backbone with fixed phi/psi angles.

Initial rotamer sets were generated using a k_B_T value of 1.5 to compute the probability of rotamer i, using the following equation: $P_{i}=\frac{e^{\left(-E_{i}/K_{b}T\right)}}{\sum_{s}e^{\left(-E_{i}/K_{b}T\right)}}$ where P is the probability, E is the rotamer energy, k_B_ is the Boltzmann constant, T is temperature and s is the rotameric state. When initially testing AutoRotLib by generating rotamer libraries for canonical amino acids and applying them to rotamer recovery tests in Rosetta it was revealed that in some cases Rosetta was not properly recovering rotamers, correct rotamers were often generated by AutoRotLib, but were not considered by Rosetta. This was due to the way in which Rosetta loads rotamers: adding rotamers until the cumulative rotamer probability is 95% (98% if buried) for a given backbone conformation or 87% (95% if buried) for semi-rotameric residues. As shown in Supplementary Table S9, increasing k_B_T to 4.0 resulted in a marked improvement in packing tests. While increasing the temperature parameter has the effect of leveling the rotamer probability across selected rotamers for a discrete backbone orientation and reduces the usefulness of any Rosetta score terms related to rotamer probability, it nevertheless produces superior results in the canonical amino acid packing test and should not diminish the greatly reduced conformational space afforded by the rotamer sampling of AutoRotLib.

The rotamer recovery test and test set are the same as described in (Pavlovicz et al., 2020). Instead of directly comparing repacked side chains to the crystallographic coordinates, we compute how well the packed rotamers fit the original electron density to remove any error or bias introduced by the crystallographers. Specifically, we use the RRComparerElecDensDiff rotamer recovery protocol in Rosetta to load the electron density maps and calculate the various correlation scores of the side chain rotamers to the density. For any side chain considered to be at the interface, as determined by the RestrictToInterface task operation (<RestrictToInterface jump = “1” distance = “8.0”>), we first determine if the correlation of the deposited coordinates are within an absolute threshold of 0.71 (Rosetta electron correlation value). If this threshold is not met, we consider this residue to be poorly resolved and discard it from the test set. If the threshold is met, then the electron density correlation difference between the deposited coordinates and the packed coordinates is computed. If this difference is less than 0.12, then the packed rotamer is considered to have successfully recovered the native packing conformation.

### Rotamer Rescoring With Quantum Mechanical Energy Calculations

Quantum Mechanical energy calculations were performed using version 5.0.1 of the ORCA package (Neese, 2012, 2018). For each NCAA, the rotamer set determined using AutoRotLib for two discrete backbone conformations (alpha: φ = −60°, ψ = −40°; beta: φ = −140°, ψ = 130°) was optimized using the B3LYP-D3BJ/6-31g* level of theory including the conductor-like polarizable continuum model (CPCM) parameterized for water to account for solvation effects (Cramer and Truhlar, 1999). The dihedral angles of all rotatable bonds between heavy atoms were constrained in order to ensure that the conformations stayed close to the input. A final energy calculation was performed at the B3LYP-D3BJ/6-311 + g** level of theory including a CPCM water solvation model. The energies are reported relative to the lowest energy conformation in kcal/mol.

### Thioether Parameterization

In order to model peptides cyclized by a thioether linkage between an acetyl group extending from the N-terminus and a downstream cysteine side chain, a patch was generated to describe the chemistry. Similar to NCAA preparation, partial charges for the linker atoms were generated using the AM1BCCELF10 charging scheme within QUACPAC (QUACPAC 2.1.2.1, 2021). Torsion constraints were also generated to better describe the geometry about the C-C-S-C bond formed from cyclization. Thioether constraints were predicted using the MMFF94S forcefield as implemented by the SZYBKI module within OpenEye on a representative piece of the linker as illustrated in Figure 2A. The linker fragment was used to characterize the dihedral energy profile about the linking bond using the MMFF94S force field within the SZYBKI (SZYBKI 2.3.0.0, 2021) module of OpenEye. To minimize the fit between the MMFF94S and Rosetta-sampled C-C-S-C torsion profiles, two periodic constraints, f_1_(x) and f_2_(x), were parameterized such that f(x) = k*(1-cos (n (x-x_o_)), f_1_: k = 0.71, *n* = 2, x_o_ = 90° and f_2_: k = 1.18, *n* = 3, x_o_ = 60°. The k values were systematically scanned to minimize the RMSD between the QM and MM energy profiles when sampled over 37 data points at regular 10° intervals. These data were built into the algorithm *thioether_macrocycle* which utilizes a GenKic-based sampling (Mandell et al., 2009; Bhardwaj et al., 2016) to build peptide backbones cyclized through a thioether bond and optimizes side chain rotamers prior to a gradient descent-based energy minimization.

### Site Saturation Mutagenesis Scanning

The peptide bound NMR and crystal structures of MCL1-PUMA (PDB 2ROC) and KDM4-CP2 (PDB 5LY1), respectively, were pre-processed for use within Rosetta as described in the Supplementary text. To estimate changes in binding energy upon mutation we used the Rosetta FastDesign (Maguire et al., 2020) protocol to systematically substitute canonical and noncanonical residues at every position within the peptide being analyzed. Changes in binding energy were calculated using the ddg mover within Rosetta and results were filtered using custom R scripts (RS Team, 2020) to determine the Δddg_calculated_ for each mutation. Since we only evaluated Δddg within Rosetta we did not calculate the unfolded state reference energies that were previously utilized for NCAA within Rosetta (Renfrew et al., 2012) as the reference term is used to calculate both the bound and unbound state energies and is canceled out by calculating the difference, or ddg, between the two states.

## Results

### Streamlining Parameterization and Rotamer Library Generation for Amino Acids in Rosetta

**FIGURE 1:**
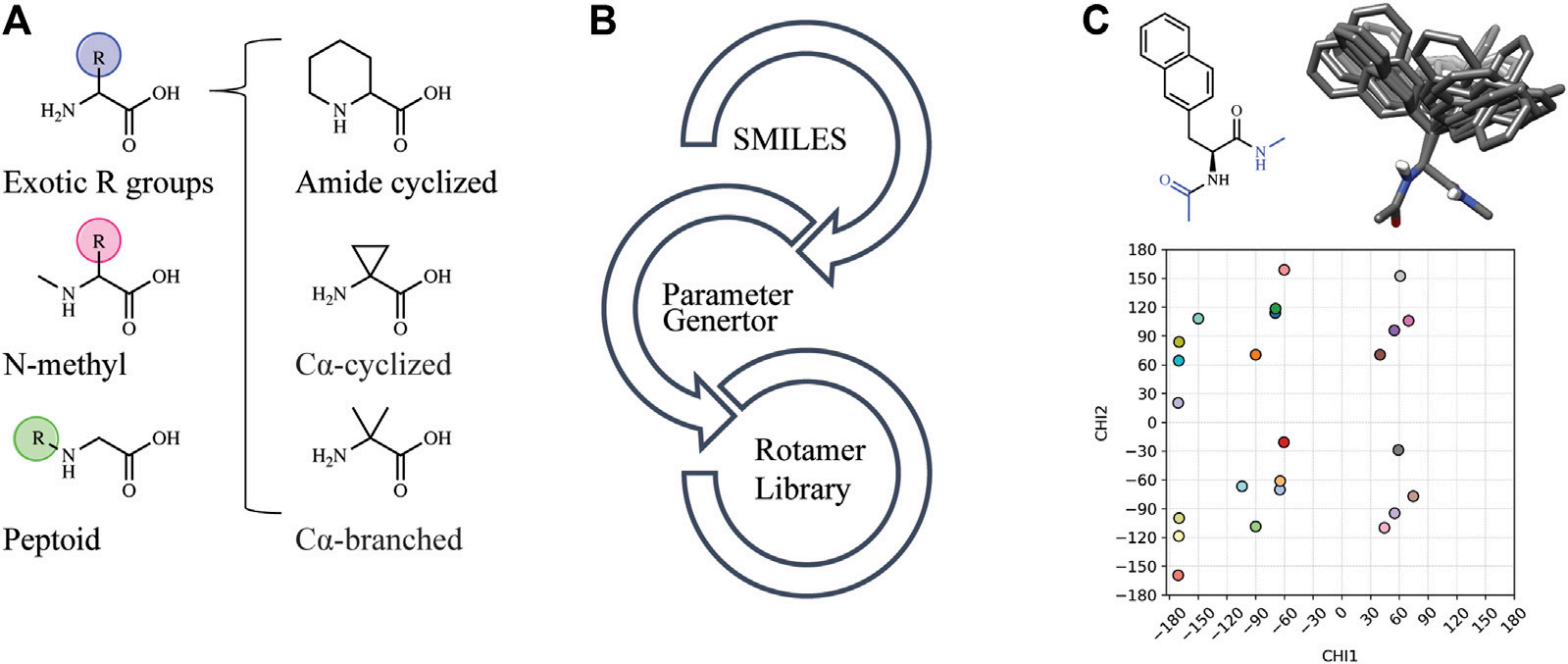
AutoRotLib was developed to parameterize chemically diverse NCAAs for Rosetta. **(A)** NCAAs that can be parameterized with our automated tools include exotic R groups, N-methyl amino acids, and peptoids with ≤4 heavy atom torsion angles. **(B)** In order to parameterize a NCAA using the automated tools, only a SMILES string and residue charge is required for generating files required for use in Rosetta. **(C)** Parameterization of the non-canonical b-(2-naphthyl)-L-alanine (2Np) using AutoRotLib requires capping the termini with acetyl and N-methyl groups (colored blue) to produces rotameric positions of the side chain for a given backbone position. Example rotamers for phi = −60°, psi = −40°; 2-D and 3-D representation (top) and 2-D representation of **X** angles (bottom) for 2Np.

Automated tools were developed to generate the necessary input files to incorporate NCAAs into Rosetta design protocols (see Methods). Currently, Rosetta requires parameterization of a NCAA in order to define atom types, connectivity, partial charges, rotatable bonds, and rotameric states. Since idealized geometries and rotamer positions for these amino acids cannot be extracted from the Protein Data Bank, as is done for canonical amino acids, these atomic-level descriptors need to be determined empirically using the previously-developed MakeRotLib protocol (Renfrew et al., 2012). Here, we sought to build upon the MakeRotLib protocol by automating the parameterization of NCAAs into Rosetta containing either exotic R groups (those found outside of the canonical 20 amino acids), N-methylated residues, or peptoids (Figure 1A). This advancement allows a user to easily incorporate a wide range of NCAAs into Rosetta. In short, parameterization using our updated workflow, AutoRotLib, only requires the user to supply a SMILES string of the NCAA and the overall charge of the side chain (Figure 1B). During parameterization, the most probable rotameric states of each side chain on a set of discretely sampled fixed backbone conformations are determined by clustering energy-minimized conformations based on their measured distance in **X**-space (Figure 1C). Unlike MakeRotLib, AutoRotLib does not require the user to specify the number or values of expected energy wells (bins) for each rotatable bond (Chi, **X**) in the side chain and thereby minimizes potential user error while also simplifying the parameterization process. Moreover, atom typing is automatically handled by OpenEye during the rotamer sampling process, further streamlining the parameterization process.

To assess AutoRotLib for production of reasonable side chain conformations, we generated rotamer libraries for the 18 canonical amino acids with flexible side chains to benchmark against the knowledge-based rotamer libraries currently used by Rosetta (Shapovalov and Dunbrack, 2011), 2011 as well as rotamer libraries generated by MakeRotLib (Renfrew et al., 2012). The generated rotamer libraries were evaluated using examples of side chain conformations from 6,950 residues found at 153 protein-protein interfaces that were repacked with Rosetta, as previously reported (Pavlovicz et al., 2020), where successful repacking was determined by calculating how well the repacked selected side chain conformation correlates to the experimental electron density. Overall, AutoRotLib performed as well as, if not slightly better than, MakeRotLib when applied to the canonical amino acid rotamer packing test as shown in Table 1. The knowledge-based Rosetta rotamer libraries showed the strongest performance in this test, which is expected given that the Rosetta rotamer libraries were trained based on a much larger set of structural data found in the Protein Data Bank (PDB), while MakeRotLib and AutoRotLib rely on empirically-determined molecular mechanics (MM) force fields. A closer look at some of the cases in which MakeRotLib and/or AutoRotlib failed to recover certain side chain conformations in our interface packing test revealed that some unrecovered side chain conformations were due to correct rotamers not being generated by the protocol, while other cases were more complex in which a cascading effect from a poorly packed neighboring side chain resulted in surrounding side chains to subsequently pack incorrectly. This may be due to missing rotamers or preferred scoring of alternate side chain combinations over the native-like solution. A more detailed analysis of this is presented in Supplementary Table S11 and Supplementary Figure S2. Outperforming knowledge-based rotamer libraries on canonical side chains was not a goal of this experiment, but the comparison remains useful as a guide.

**TABLE 1:** Rotamer recovery of canonical amino acids after repacking with the Rosetta standard libraries (dun10 with Shapovalov’s corrections) and libraries generated by the MakeRotLib and AutoRotLib protocols using Rosetta 3.10 and the REF2015 score function. Rotamer recovery reported as the average ±standard deviation for three separate calculations.

Amino acid	Rotamer recovery (%)		
	Rosetta baseline	MakeRotLib	AutoRotLib
L-cysteine	96.6 ± 0.0	83.6 ± 0.8	80.2 ± 0.8
L-serine	97.7 ± 0.0	90.8 ± 0.2	91.1 ± 0.3
L-threonine	98.7 ± 0.1	93.5 ± 0.3	94.5 ± 0.3
L-valine	99.6 ± 0.0	97.7 ± 0.2	99.5 ± 0.1
L-leucine	96.3 ± 0.1	89.2 ± 0.4	93.4 ± 0.2
L-isoleucine	98.2 ± 0.0	95.2 ± 0.1	95.8 ± 0.2
L-methionine	84.8 ± 0.2	50.5 ± 0.4	73.1 ± 0.5
L-arginine	63.5 ± 0.4	39.8 ± 0.5	45.7 ± 0.6
L-lysine	86.3 ± 0.4	76.2 ± 0.3	76.6 ± 0.2
L-proline	99.8 ± 0.0	99.8 ± 0.0^a^	99.4 ± 0.1
L-asparagine	94.2 ± 0.1	80.3 ± 0.3	81.4 ± 0.1
L-aspartic acid	90.8 ± 0.3	75.4 ± 0.1	78.7 ± 0.4
L-glutamine	81.7 ± 0.4	67.4 ± 0.5	77.3 ± 0.1
L-glutamic acid	79.1 ± 0.2	66.6 ± 0.9	72.8 ± 0.4
L-histidine	91.5 ± 0.0	71.6 ± 0.0	71.0 ± 0.6
L-phenylalanine	94.1 ± 0.1	88.1 ± 0.7	91.0 ± 0.7
L-tryptophan	86.8 ± 0.3	75.8 ± 0.6	81.4 ± 1.1
L-tyrosine	87.6 ± 0.2	77.0 ± 0.8	84.6 ± 0.4

^a^Proline uses the Dunbrack rotamer library in the MakeRotLib test due to difficulty parameterizing the cyclic amino acid.

Given the ability of AutoRotLib to reproduce rotamer conformations of canonical amino acids, we next evaluated AutoRotLib for use with NCAAs composed of exotic R groups, N-methylation, and peptoids. Unfortunately, most NCAAs are either underrepresented or completely absent from the PDB, making it difficult to benchmark NCAA rotamers against experimental data. Considering that Rosetta only loads a subset of the most probable rotamers from the rotamer library—the cumulative top 87% (or 95% if buried)—for a semi rotameric sidechain at a given backbone conformation, we elected to compare the rotamers generated by AutoRotLib for several NCAAs and rescore those rotamers using quantum mechanics (QM) by focusing on a small library of 6 NCAAs (Table 2) composed of different classes of NCAAs (exotic alpha, N-methyl, and peptoid). Specifically, we compared the rotamer energies generated by AutoRotLib to QM at two different backbone conformations for each NCAA: an idealized ɑ-helix and β-sheet conformation. To rescore rotamers by QM, rotamer conformations were first minimized with restrained heavy-atom dihedral angles. Once the QM energies were determined for each rotamer, the rotamers were rank-ordered and compared to the AutoRotLib rotamers at the same backbone conformation (Table 2 and Supplementary Tables S1–S6). In general, there was excellent agreement between AutoRotLib and QM calculated energies (Table 2). For residues like 2Np (Supplementary Table S1), 2Th (Supplementary Table S2), PeG (Supplementary Table S3), and MeF (Supplementary Table S5), subtle differences were observed between MM and QM rank ordering of rotameric states, especially at high energy conformations. Considering that the high energy rotamers would likely not be critical for Rosetta design and given the general agreement between the relative energies for each rotamer calculated with the MM and QM metrics in Table 2, we anticipate NCAAs parameterized with AutoRotLib are most likely useful for protein design purposes.

**TABLE 2:** NCAAs generated by AutoRotLib were scored using a MM force field and rescored using QM.

Noncanonical	Rotamer agreement between MM and QM				
	2D structure	phi	psi	87% of library	95% of library
b-(2-Naphthyl)-L-alanine (2Np)	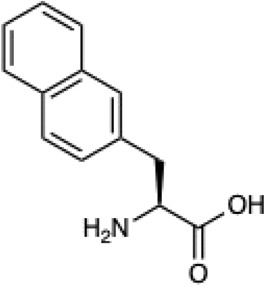	−60	−40	14 of 16	19 of 19
		−110	130	14 of 17	19 of 21
L-2-thienyl-Ala (2Th)	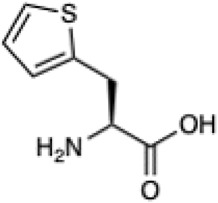	−60	−40	9 of 10	10 of 11
		−110	130	9 of 10	10 of 11
N-a-Methyl-L-phenylalanine (MeF)	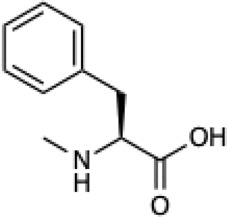	−60	−40	7 of 7	7 of 8
		−110	130	6 of 7	9 of 9
N-a-Methyl-L-histidine (MeH)	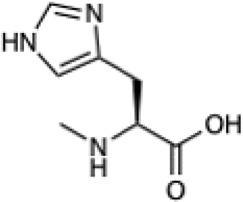	−60	−40	19 of 19	21 of 21
		−110	130	17 of 17	21 of 21
N-(2-Phenylethyl)-glycine (PeG)	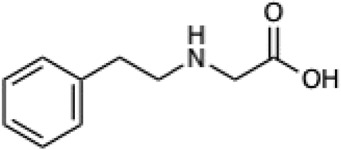	−60	−40	13 of 14	16 of 16
		−110	130	11 of 12	15 of 16
Cyclopropyl-methyl-glycine (CpG)	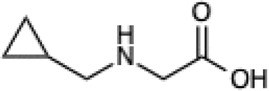	−60	−40	5 of 5	6 of 6
		−110	130	5 of 5	6 of 6

### Incorporation of Thioether-Based Cyclization

**FIGURE 2:**
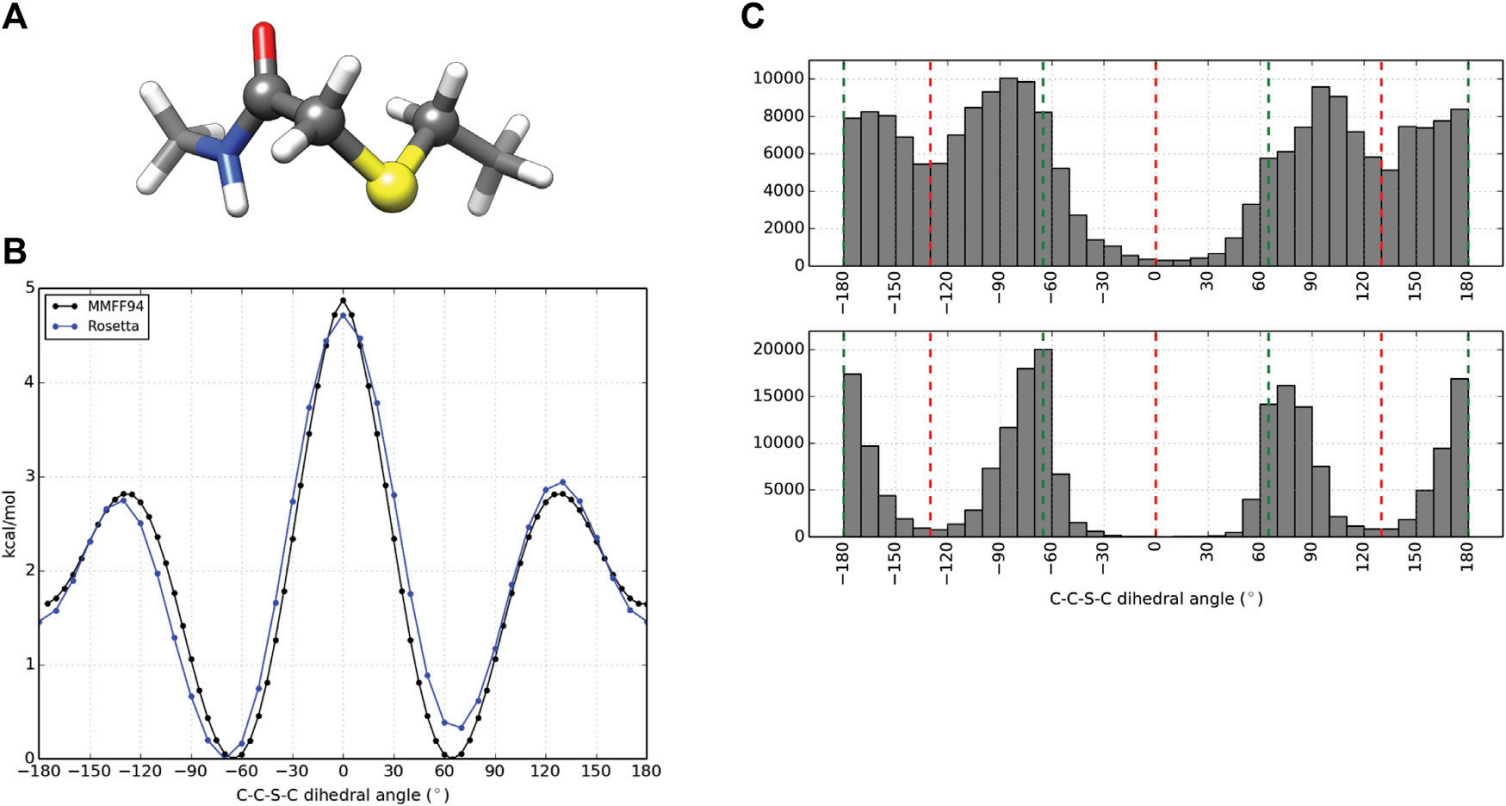
Parameterization and application of thioether linker. **(A)** Representative molecule used for thioether parameterization. The angle scanned for dihedral parameterization is highlighted by larger spheres. **(B)** C-C-S-C torsional profiles by the MMFF94S force field (black) and Rosetta (blue) after dihedral constraints were optimized. **(C)** C-C-S-C angle distributions for 100,000 thioether-linked 8-mer (AAAAAAAC) macrocycles generated without additional dihedral constraints (top) and with dihedral constraints applied (bottom). The red dashed lines represent the theoretical maxima of the dihedral energy landscape, while the green lines represent the theoretical minima for the thioether bond. Note that each thioether bond has two C-C-S-C torsions.

Computational design and evaluation of thioether-cyclized macrocycles were accomplished in Rosetta by chemically modifying the N-terminal residue to add the appropriate acetyl atoms required for closure of the macrocycle with a C-terminal thiol group most commonly originating from a cysteine residue. Additional torsional constraints were added to better describe the geometry about the C-C-S-C bond formed from cyclization of the peptide. A representative piece of the thioether linker (Figure 2A, Supplementary Figure S4) was used to measure the energy barrier about the C-S bonds using the MMFF94S (MM) force field as implemented by the SZYBKI module of OpenEye tools (SZYBKI 2.3.0.0, 2021). With the application of two periodic constraints, the MM and Rosetta-sampled C-C-S-C torsional profiles were minimized to an RMSD of 0.2325 Å when measured over 37 data points sampled at regular 10° intervals (Figure 2B). To test the torsional constraints of the thioether linker we wrote an application in Rosetta, *thioether_macrocycle*, to generate three-dimensional models from a primary sequence. The aforementioned dihedral constraints are applied during energy minimization of the predicted macrocycle structure. As shown in Figure 2C, this results in conformer populations that cluster around the expected energy minima of −60°, 60°, and 180° for the C-C-S-C bonds that define the thioether linkage. Without the constraints, the C-C-S-C dihedral angles of the predicted macrocycle structures were more evenly distributed from ±60–180° (2C, upper), with an overpopulation of structures occupying a linker torsional space near the theoretical energy maxima of ±130°. Together, the structure prediction application with the torsional corrections demonstrates that the thioether linkage can be modeled within Rosetta.

### Parameterizing a Library for *in silico* Site Saturation Mutagenesis

**FIGURE 3:**
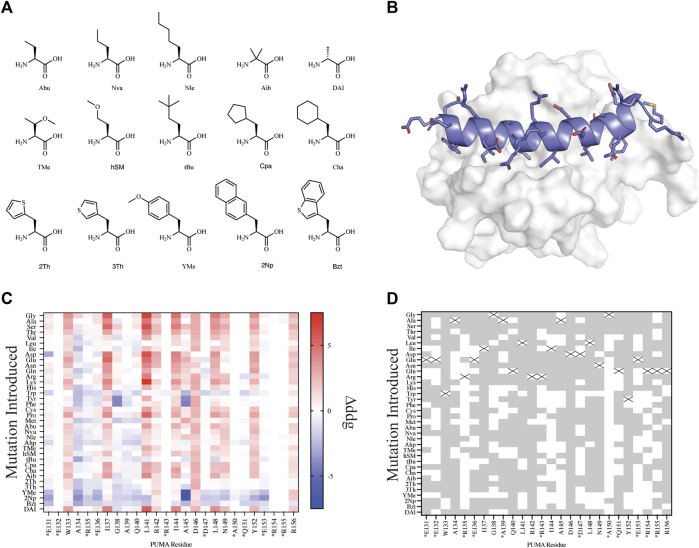
Mutational scanning of canonical and NCAA on the peptide PUMA bound to MCL-1. **(A)** NCAA shown in stick format were evaluated and parameterized with AutoRotLib. The NCAA DAI was previously parameterized in Rosetta as DAL. **(B)** PUMA peptide depicted in cartoon format and colored blue is bound to MCL-1 (PDB 2ROC). **(C)** Heatmap analysis of Δddg_calculated_ (ddg_wt_−ddg_design_) for individual residues. Native residues that are >75% solvent exposed are indicated by an asterisk*. **(D)** Agreement between Δddg_calculated_ and ΔΔG_binding_ for point mutations to be stabilizing or destabilizing on PUMA peptide shown as grey tiles and point mutations that differed between the two datasets are shown as white tiles. Tiles that are marked with an X represent the native residue.

In order to test our automated workflows for computational design with NCAAs, we parameterized a small library of 15 NCAAs using AutoRotLib (Figure 3A). The library was curated from a list of 21 NCAAs previously reported for *in vitro* site saturation mutagenesis (Rogers et al., 2018) using the RaPID platform. In total, 6 of the 21 NCAAs previously reported (Rogers et al., 2018) were not included in our parameterized library because they either contained >4 heavy atom torsional angles or were not alpha amino acids. Currently, AutoRotLib is limited to parameterizing NCAAs containing side chains with ≤4 heavy atom torsion angles due to limitations in the backbone-dependent rotamer handling code in Rosetta. Additionally, Rosetta design protocols currently cannot interconvert between different classes of amino acids, like N-methyl/peptoids from alpha amino acids. As a result, we can only evaluate NCAA designs with ≤4 heavy atom torsion angles that are within the same amino acid class as the parent residue.

To better understand differences between rotamer libraries generated with AutoRotLib or derived from the Dunbrack Library we also evaluated our site saturation mutagenesis protocols using the different rotamer libraries as inputs for canonical amino acids (Supplementary Figure S3). While similar, with native-like rotamers found in all of these libraries, there are slight conformational differences among them. These differences can impact the pairwise scoring of the side chain in question depending on the environment in which it is being packed. As observed in the analysis of the interface packing benchmark, these conformational differences can result in scoring differences that prioritize the selection to favor one mutation over another.

### Evaluating a Peptide Antagonist

Since we are interested in using design protocols to engineer and screen novel side chains that improve peptide interactions with a target protein, we elected to perform site saturation mutagenesis studies by sequentially screening every amino acid within our parameterized library at each residue position using FastDesign (Maguire et al., 2020) on the peptide PUMA using the ternary complex of PUMA:MCL-1 (PDB 2ROC, Figure 3). PUMA was used as a case study as it has been extensively screened and evaluated for incorporation of canonical and noncanonical amino acids (Day et al., 2008; Rogers et al., 2014, 2018). To understand how our *in silico* predictions compared to previous mutagenesis studies we first calculated the per residue Δddg_calculated_ (ddg_wt_−ddg_design_) for each design (Figure 3C) and compared the Δddg_calculated_ to the previously reported change in binding free energy (ΔΔG_binding_) that was extrapolated from a selection-based experiment (Rogers et al., 2018). Since neither the Δddg_calculated_ nor the experimental ΔΔG_binding_ are absolute values, we took a binary approach to characterize mutations as either stabilizing (Δddg_calculated_ < 0 and ΔΔG_binding_ < 0) or destabilizing (Δddg_calculated_ > 0 and ΔΔG_binding_ > 0). Mutations that were found to be stabilizing or destabilizing in both the previously reported experimental data and our *in silico* data were considered in agreement. For the purpose of evaluating *in silico* design accuracy, this type of binary analysis has been used previously (Nguyen et al., 2019). Overall, we observed varying agreement at residue positions between the two datasets ranging from 16.7 to 97.2% agreement for the mutations evaluated. Similarly, if we decouple NCAA from CAA in our analysis of point mutations, we found that canonical amino acids have a greater agreement measured at 20 or 26 residue positions compared to NCAA (Supplementary Figure S3). For designs at residue positions with low agreement to the experimental dataset, like residue positions R135 and A150, we noticed these residues were mostly solvent exposed (Figure 3D and Supplementary Table S10). Considering the ddg calculation is an estimate of interface interaction it is not surprising that solvent exposed designs would be more challenging to evaluate by the ddg metric within Rosetta. Therefore, these could be excellent positions to modulate peptide pharmacokinetic properties through introduction of new side chains without affecting the peptide binding properties. We also compared our *in silico* results to previously published (Rogers et al., 2018) biophysical data (Supplementary Table S7) and found of the 29 published mutations 22 mutations were destabilizing (Δddg_calculated_ > 0 and K_d,mutant_ > K_d,wt_) in both datasets, and 2 mutations were stabilizing (Δddg_calculated_ < 0 and K_d,mutant_ < K_d,wt_) in both datasets (Supplementary Table S7). Importantly, if we filter the data further and remove Δddg_calculated_ values between −1 and +1, as these are considered within the noise variance of the ddg calculation (Barlow et al., 2018), the agreement between experiments improves to 14 out of 16 mutations (Supplementary Table S7). The two outliers that were not in agreement between the filtered data sets include Tyr152Bzt and Ala144Cha. Upon closer inspection of the design outliers, two observations were made that should be considered for assessing the accuracy of a design: 1) changes in the side chain size affect the number of residue contacts used to calculate a per residue ddg and 2) design on buried residues, like Ala144, might induce an alternative binding mode that cannot be modeled accurately by the FastDesign protocol. This is not surprising considering that the FastDesign protocol is optimized for small perturbations of the side chain and does not sufficiently sample small-to-large substitutions at an interface (Maguire et al., 2020).

### Evaluating a Thioether-Linked Macrocycle Antagonist

**FIGURE 4:**
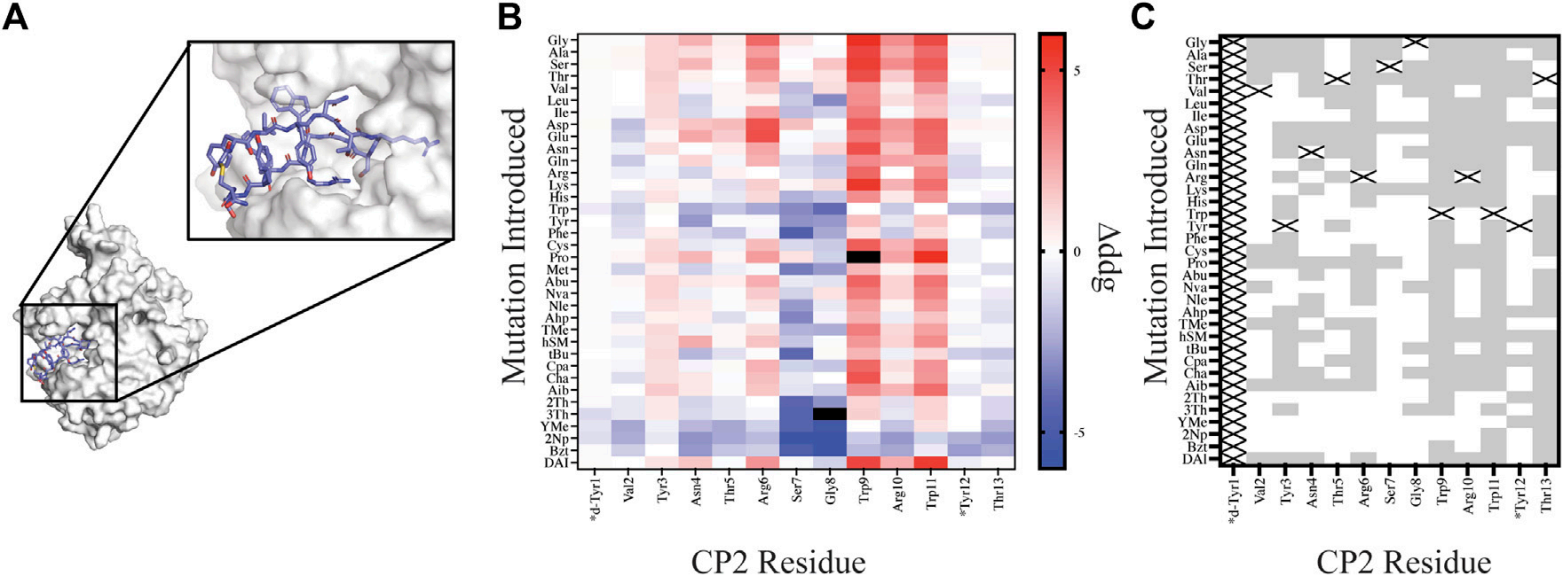
Site saturation mutagenesis on Macrocycle CP2 bound to target KDM4. **(A)** The crystal structure of CP2-KDM4 (PDB 5LY1) was used as the initial model for site saturation mutagenesis. **(B)** Heat map of Δddg_calculated_ values for all mutations evaluated. Tiles that are colored black were found to have a Δddg_calculated_ > 5. Native residues that are >75% solvent exposed are indicated by an asterisk*. **(C)** Agreement measured between free energy of mutations calculated for designs using Δddg_calculated_ and previously reported ΔΔG_binding_ values (Rogers et al., 2018) displayed as grey tiles with white tiles representing differences between Δddg_calculated_ and ΔΔG_binding_ values. Tiles that are marked with an X represent the native residue.

To design NCAAs within a thioether-linked peptide macrocycle, we also tested our automated workflows on the macrocycle CP2 bound to KDM4 (Figure 4A, PDB 5LY1). CP2 was first identified from an mRNA display screen using the RaPID platform (Kawamura et al., 2017) and is cyclized through a thioether bond between an N-terminal acetyl group on D-Tyr1 and the thiol group of Cys14. Using the crystal structure of CP2 bound to KDM4, we screened our library of natural amino acids and AutoRotLib parameterized NCAAs for all possible CP2 single point mutations at positions 1–13. Point mutations at position 14 were not considered in order to preserve the thiol group required for cyclization. One distinct advantage of *in silico* screening over the RaPID platform for thioether macrocycles is that position 1 can be mutated during design, unlike the RaPID system which is dependent on *in vitro* translation with only one initiator tRNA. *In vitro* screening of additional residues at position 1 using the RaPID platform therefore requires generating multiple libraries with different initiators encoded on the initiator tRNA. Hence, our ability to evaluate multiple amino acids *in silico*, especially at position 1, could help inform design of new libraries and reduce the number of libraries needed to probe diversity *in vitro*.

In order to compare our *in silico* designs on CP2 to previous mutagenesis binding studies we calculated the number of mutations that were found to be stabilizing (Δddg_calculated_ < 0 and ΔΔG_binding_ < 0) and destabilizing (Δddg_calculated_ > 0 and ΔΔG_binding_ > 0) between the two datasets using the Δddg_calculated_ and the experimental ΔΔG_binding_ values (Rogers et al., 2018). From our comparative analysis we found the mutational effects on binding energy range between the two datasets from 17% agreement to 94% agreement, with 7 of the 12 residue positions (Position 1 and Position 14 not compared) having >50% agreement. Moreover, if we separate NCAA from the canonical amino acids in our analysis of point mutations, we found that CAA have a greater agreement at 11 of the 12 residue positions evaluated then the NCAA (Supplementary Figure S3). For multiple designs at position Ser7 and Gly8 the mutation was predicted to stabilize the ternary complex based on a per residue Δddg_calculated_ < 0; however, the design significantly altered the backbone conformation of the macrocycle, based on calculating the RMSD of the design relative to the native structure, rendering a likely inaccurate binding pose. Assuming that the backbone conformation is critical for target engagement (Appavoo et al., 2019), we then filtered our designs using two RMSD filters: 1) RMSD_macrocycle_ was calculated using the heavy atoms of the CP2 backbone residues and 2) RMSD_interface_ was calculated from the interface residues also using the backbone heavy atoms. The RMSD filters, were set at 0.3 Å and 0.5 Å for RMSD_macrocycle_ and RMSD_interface_, respectively, as these values were double the median RMSD for relaxed structures with the native sequence. Application of these stringent filters to our dataset of 420 point mutations on CP2 removed 183 designs; 65 of which were not in agreement with the *in vitro* dataset. Despite the benefit of these additional filters for assessing macrocycle designs, predictive modeling of side chains that stabilize a binding interface remains challenging and not always accurate, as reported elsewhere (Nguyen et al., 2019; Mulligan et al., 2020; Hosseinzadeh et al., 2021). This is in part because macrocycles are dynamic molecules that are sensitive to small perturbations which may alter backbone and side chain conformations. In order to successfully narrow down the massive chemical space that should be considered for macrocycle optimization, experiment and computation will need to be combined to identify residue positions that are mutable, residue positions that should remain unchanged, and fine-tune the NCAA chemical space that should be further evaluated to accelerate empirical discovery and optimization of macrocycles.

## Discussion

In recent years substantial advancements have been made towards the development and design of peptide therapeutics that contain NCAAs. Here, we built upon the strategies initially developed by Renfrew et al. (2012) to simplify and automate NCAA parameterization for use in the design program Rosetta. This updated protocol, reported herein, incorporates tools from OpenEye to build rotamer libraries that were validated by sampling experiments at protein-protein interfaces and by QM energy calculations. With the ability to quickly parameterize NCAAs that include exotic R groups, peptoids, or N-methylation, we can now more easily screen NCAAs *in silico* to inform *in vitro* selection experiments and generate new hypotheses that would otherwise not be feasible by looking at a simple peptide-target structure.

As part of our effort to accelerate *in silico* design using NCAAs in Rosetta, we also developed new tools to design on thioether based macrocycles. Thioether based cyclization has the distinct advantage over other cyclization strategies in that it is an efficient and spontaneous cyclization chemistry that is amenable to an *in vitro* translation setting such as the RaPID system. Moreover, NCAA-containing macrocycles discovered on the RaPID platform are demonstrated as potent target binders (Kawamura et al., 2017; Katoh et al., 2020). While the RaPID platform is a powerful tool for screening and optimizing macrocycles, the addition of NCAAs into designs presents a challenge given the vast chemical space available to NCAAs and the limitations on the number of AAs that may be encoded in any single RaPID library. Here, the use of *in silico* prescreening could be used to focus a library of NCAAs that should be considered for *in vitro* experiments. Together, these approaches have the potential to rapidly enable affinity maturation and pharmacokinetic optimization of peptide macrocycles identified from combinatorial libraries.

Considering the utility of being able to rapidly screen a diverse chemical space *in silico* it is also important to recognize some of the current limitations for incorporating NCAAs into Rosetta. First, the Ramachandran space that is sampled by a NCAA *a priori* is unknown. Certainly, this will become an issue of less concern as the field of NCAAs continues to expand and new tools are developed for modeling NCAA behavior *in silico*, such as molecular dynamics (Khoury et al., 2014). Nonetheless, we currently have an incomplete understanding of how a NCAA might alter the backbone of the peptide scaffold or the conformational landscape of the unbound peptide. Continued development of design protocols that stabilize the bound conformation, either through cross linking or mutagenesis, have the potential to both decrease the entropic penalty of binding and preserve the critical interactions that are observed within the macrocycle bound structure. Second, predictive modeling of side chains that stabilize a binding interface, including both the sampling and scoring aspects, remains an outstanding problem with discrepancies often reported between calculated ddg’s and experimentally determined values (Nguyen et al., 2019; Hosseinzadeh et al., 2021; Mulligan et al., 2021). This can lead to false positive or false negative interpretations of designs as was observed here for the PUMA and CP2 designs. Additional design metrics, enhanced sampling, and new scoring methods like Rosetta’s genpot score function, under active development, that introduces additional physics-based score terms need to be considered to improve designs with NCAAs. This may require developing new methods that appropriately define the Rosetta reference energy values for each NCAA to properly compensate for the sometimes-outsized attractive van der Waals score component possible for very large side chains. To address concerns with the reference energy term for NCAA’s previous studies have introduced the modifiable reference energy for NCAA (Renfrew et al., 2012) and the amino acid composition score term (Mulligan et al., 2021). By enlisting site saturation mutagenesis to probe chemical space and ddg measurements to evaluate binding, the contribution of the reference term to the binding pose is negligible. Nonetheless, methods like *in silico* saturation mutagenesis are still limiting from a design perspective as the user is unable to identify compensatory mutations without a well-established reference energy for each NCAA and incorporation of the reference energy will be necessary for complex design strategies. Despite these challenges, continued development on the energy score, NCAA Ramachandran sampling, design filters, and design algorithms will significantly improve our ability to accurately design peptides and macrocycles with NCAAs in Rosetta.

We anticipate tools like AutoRotLib will be useful for peptide engineers to quickly parameterize NCAAs so that they may be incorporated into a peptide design. In doing so, a user will be able to evaluate a variety of side chain isomers, bioisosteres, and structural analogs at individual residue positions to improve the binding interactions and pharmacokinetics of a peptide. Coupled with technologies like the RaPID platform, computational mutational scanning will be a critical step informing the design of new libraries that can be screened empirically to help optimize the potency and properties of peptide therapeutics.

## Resource Identification Initiative

Rosetta, RRID:SCR_015701

## Data Availability

The algorithms for thioether_macrocycle and score_thioether_macrocycle have been implemented as a Rosetta protocol. Python scripts to generate rotamer libraries with AutoRotLib have been uploaded to https://github.com/rpavlovicz/AutoRotLib. The scripts require a license for the following OpenEye modules: OEChem, Omega, Quacpac, and Szybki.
