# Supplementary material for: Computational Site Saturation Mutagenesis of Canonical and Non-Canonical Amino Acids to Probe Protein-Peptide Interactions
Source: Front Mol Biosci. 2022 Apr 14;9:848689. doi: 10.3389/fmolb.2022.848689 (PMC9047896; doi:10.3389/fmolb.2022.848689)
Supplement: Supplementary file 1 [file DataSheet1.docx]

Supplementary Material

Table S1. Rotamer library at a given phi/psi angle generated using AutoRotLib for b-(2-Naphthyl)-L-alanine (2Np). The change in energy (ΔE) was calculated from the difference between the lowest energy rotamer conformation at a given phi/psi using MMFF94S and QM separately.

| phi (deg) | psi (deg) | chi 1 | chi 2 | Probability (%) | ΔE (MMFF94S, kcal/mol) | ΔE (QM, kcal/mol) |
| --- | --- | --- | --- | --- | --- | --- |
| -60 | -40 | 60.11 | -99.83 | 8.71 | 0.00 | 0.30 |
|  |  | 60.01 | 83.48 | 8.62 | 0.04 | 0.00 |
|  |  | 59.85 | -118.05 | 7.84 | 0.42 | 0.77 |
|  |  | 59.91 | 64.76 | 7.58 | 0.55 | 0.24 |
|  |  | 79.67 | 108.02 | 6.24 | 1.33 | 1.81 |
|  |  | 74.85 | -67.95 | 6.07 | 1.44 | 1.65 |
|  |  | 150.10 | -80.22 | 5.47 | 1.86 | 2.66 |
|  |  | 59.04 | -158.56 | 5.11 | 2.13 | 1.92 |
|  |  | 150.11 | 103.68 | 5.02 | 2.20 | 2.76 |
|  |  | 59.54 | 21.38 | 4.96 | 2.25 | 2.63 |
|  |  | 159.89 | -66.40 | 4.92 | 2.28 | 2.40 |
|  |  | 155.13 | 117.77 | 4.67 | 2.49 | 2.45 |
|  |  | 139.70 | -112.96 | 4.29 | 2.83 | 3.57 |
|  |  | 139.79 | 65.40 | 3.89 | 3.22 | 3.41 |
|  |  | 149.65 | 21.25 | 3.23 | 3.97 | 4.43 |
|  |  | 149.17 | -159.37 | 3.20 | 4.01 | 3.72 |
|  |  | 21.25 | -110.82 | 2.19 | 5.52 | 2.76 |
|  |  | 16.09 | 73.45 | 1.84 | 6.23 | 2.75 |
|  |  | -54.62 | -85.37 | 1.55 | 6.91 | 3.68 |
|  |  | -54.52 | 106.87 | 1.34 | 7.50 | 4.76 |
|  |  | -49.87 | 111.80 | 1.30 | 7.62 | 5.51 |
|  |  | -50.43 | -68.61 | 1.20 | 7.93 | 4.97 |
|  |  | -130.11 | -98.81 | 0.41 | 12.19 | 9.38 |
|  |  | -76.19 | -17.80 | 0.37 | 12.67 | 9.87 |
| -110 | 130 | 169.85 | -41.55 | 9.38 | 0.00 | 0.00 |
|  |  | 169.62 | 153.47 | 8.82 | 0.24 | 0.42 |
|  |  | 169.51 | 158.26 | 8.72 | 0.29 | 0.51 |
|  |  | 168.75 | -17.60 | 8.08 | 0.59 | 1.25 |
|  |  | 160.53 | -60.44 | 8.05 | 0.61 | 0.22 |
|  |  | 155.73 | 119.43 | 6.09 | 1.73 | 1.20 |
|  |  | 75.19 | -129.69 | 4.02 | 3.39 | 2.12 |
|  |  | 75.22 | 53.65 | 4.00 | 3.41 | 2.17 |
|  |  | 153.78 | 17.48 | 3.77 | 3.64 | 2.92 |
|  |  | 154.55 | -160.35 | 3.70 | 3.72 | 2.05 |
|  |  | 69.26 | -175.42 | 3.68 | 3.73 | 1.73 |
|  |  | 72.33 | 1.81 | 3.64 | 3.78 | 2.22 |
|  |  | 80.08 | 68.46 | 3.46 | 3.99 | 2.67 |
|  |  | 84.94 | -111.77 | 3.21 | 4.29 | 3.16 |
|  |  | -76.25 | -170.23 | 3.05 | 4.49 | 2.02 |
|  |  | 75.51 | -22.24 | 2.92 | 4.66 | 3.50 |
|  |  | 78.83 | 156.24 | 2.85 | 4.76 | 2.63 |
|  |  | -80.68 | -154.63 | 2.54 | 5.23 | 1.76 |
|  |  | -77.35 | 16.31 | 2.51 | 5.26 | 2.49 |
|  |  | -78.32 | 23.56 | 2.44 | 5.39 | 1.97 |
|  |  | -66.18 | -12.68 | 1.68 | 6.87 | 3.81 |
|  |  | -68.29 | 168.46 | 1.41 | 7.58 | 2.80 |
|  |  | -95.97 | 59.84 | 1.02 | 8.87 | 3.43 |
|  |  | -100.03 | -118.63 | 0.97 | 9.06 | 4.75 |

Table S2. Rotamer library at a given phi/psi angle generated using AutoRotlib for L-2-thienyl-Ala (2Th). The change in energy (ΔE) was calculated from the difference between the lowest energy rotamer conformation at a given phi/psi using MMFF94S and QM separately.

| phi (deg) | psi (deg) | chi 1 | chi 2 | Probability (%) | ΔE (MMFF94S, kcal/mol) | ΔE (QM, kcal/mol) |
| --- | --- | --- | --- | --- | --- | --- |
| -60 | -40 | -69.46 | -85.81 | 13.97 | 0.00 | 0.78 |
|  |  | 55.30 | 95.27 | 12.39 | 0.48 | 0.77 |
|  |  | -65.02 | 100.83 | 10.64 | 1.09 | 0.00 |
|  |  | -64.31 | -47.11 | 10.50 | 1.14 | 0.86 |
|  |  | -165.09 | -90.85 | 8.12 | 2.17 | 1.28 |
|  |  | 55.41 | -84.74 | 7.95 | 2.25 | 0.35 |
|  |  | -170.08 | 70.46 | 7.49 | 2.49 | 0.95 |
|  |  | -60.02 | 140.98 | 7.37 | 2.56 | 0.40 |
|  |  | 60.65 | 135.41 | 6.27 | 3.20 | 2.57 |
|  |  | -170.90 | 39.60 | 6.24 | 3.22 | 2.27 |
|  |  | -161.68 | -127.54 | 5.34 | 3.84 | 1.34 |
|  |  | 68.45 | -43.98 | 3.73 | 5.28 | 2.98 |
| -110 | 130 | -174.84 | -99.94 | 11.85 | 0.00 | 0.00 |
|  |  | -164.98 | 79.22 | 11.65 | 0.07 | 0.61 |
|  |  | -74.54 | 94.52 | 10.13 | 0.62 | 0.93 |
|  |  | -69.56 | -75.88 | 9.30 | 0.97 | 0.75 |
|  |  | -170.63 | 50.09 | 8.92 | 1.13 | 1.14 |
|  |  | -175.26 | -133.97 | 8.31 | 1.42 | 0.79 |
|  |  | 55.01 | 90.33 | 8.21 | 1.46 | 1.68 |
|  |  | 55.41 | -77.68 | 7.85 | 1.64 | 1.65 |
|  |  | -69.50 | -47.51 | 7.80 | 1.67 | 2.65 |
|  |  | -78.86 | 123.36 | 7.07 | 2.06 | 1.82 |
|  |  | 65.64 | -40.02 | 4.47 | 3.90 | 4.57 |
|  |  | 65.21 | 133.52 | 4.43 | 3.93 | 2.29 |

Table S3. Rotamer library at a given phi/psi angle generated using AutoRotLib for N-(2-Phenylethyl)-glycine (PeG) with the omega angle fixed at 180 degrees (deg). The change in energy (ΔE) was calculated from the difference between the lowest energy rotamer conformation at a given phi/psi using MMFF94S and QM separately.

| phi (deg) | psi (deg) | chi 1 | chi 2 | chi 3 | Probability (%) | ΔE (MMFF94S, kcal/mol) | ΔE (QM, kcal/mol) |
| --- | --- | --- | --- | --- | --- | --- | --- |
| -60.00 | -40.00 | 82.55 | 177.68 | 84.68 | 9.32 | 0.00 | 0.70 |
|  |  | 80.41 | 176.27 | 59.90 | 8.12 | 0.54 | 1.29 |
|  |  | 81.58 | -177.33 | 122.87 | 7.59 | 0.82 | 1.43 |
|  |  | 89.07 | -70.75 | 112.37 | 6.93 | 1.18 | 0.83 |
|  |  | 62.46 | 72.22 | 91.93 | 6.91 | 1.19 | 0.00 |
|  |  | -87.49 | -179.08 | 87.78 | 6.65 | 1.35 | 1.08 |
|  |  | 88.63 | -70.46 | 125.83 | 6.15 | 1.65 | 1.27 |
|  |  | -89.54 | 179.82 | 63.39 | 5.83 | 1.87 | 1.49 |
|  |  | -84.28 | -176.94 | 122.94 | 5.63 | 2.01 | 1.86 |
|  |  | -63.32 | -70.70 | 120.42 | 5.20 | 2.33 | 1.28 |
|  |  | -76.82 | -85.27 | 114.86 | 5.00 | 2.49 | 2.12 |
|  |  | 62.28 | 69.92 | 57.88 | 4.95 | 2.53 | 1.17 |
|  |  | -91.97 | 77.10 | 56.40 | 4.82 | 2.63 | 1.97 |
|  |  | 58.75 | 78.63 | 121.48 | 4.73 | 2.71 | 2.56 |
|  |  | -91.38 | 79.40 | 60.32 | 4.60 | 2.82 | 2.11 |
|  |  | 73.08 | -138.91 | 52.15 | 3.15 | 4.33 | 3.96 |
|  |  | -103.26 | 121.09 | 111.41 | 2.28 | 5.63 | 5.15 |
|  |  | -61.34 | -105.35 | 59.15 | 2.17 | 5.83 | 4.99 |
| -110.00 | 130.00 | -111.06 | -177.96 | 94.89 | 9.80 | 0.00 | 1.22 |
|  |  | 56.07 | 178.56 | 80.15 | 9.67 | 0.05 | 0.39 |
|  |  | 58.37 | 75.80 | 88.92 | 9.07 | 0.31 | 0.00 |
|  |  | 52.99 | 177.15 | 56.98 | 8.71 | 0.47 | 0.98 |
|  |  | -113.26 | -174.17 | 120.97 | 7.51 | 1.06 | 1.79 |
|  |  | -113.06 | -178.52 | 56.98 | 7.31 | 1.17 | 1.76 |
|  |  | 55.19 | -177.44 | 120.00 | 7.07 | 1.30 | 0.83 |
|  |  | 46.04 | 59.84 | 59.37 | 6.80 | 1.46 | 0.69 |
|  |  | 46.04 | 77.25 | 118.65 | 6.79 | 1.47 | 1.92 |
|  |  | -104.32 | -133.48 | 128.16 | 5.49 | 2.31 | 3.61 |
|  |  | 76.47 | -74.14 | 126.10 | 4.75 | 2.90 | 1.58 |
|  |  | 87.89 | -71.64 | 115.65 | 4.68 | 2.96 | 1.13 |
|  |  | 76.56 | -136.33 | 61.43 | 2.98 | 4.76 | 3.28 |
|  |  | -119.54 | 48.27 | 104.78 | 2.19 | 6.00 | 2.37 |
|  |  | -119.02 | 47.64 | 115.96 | 2.14 | 6.09 | 2.59 |
|  |  | -100.03 | -81.75 | 117.00 | 1.88 | 6.60 | 2.84 |
|  |  | -104.59 | 137.94 | 67.77 | 1.81 | 6.76 | 4.24 |
|  |  | -101.40 | -34.47 | 74.16 | 1.37 | 7.88 | 3.18 |

Table S4. Rotamer library at a given phi/psi angle generated using AutoRotlib for cyclopropyl-methyl-glycine (CpG) with the omega angle fixed at 180 degrees (deg). The change in energy (ΔE) was calculated from the difference between the lowest energy rotamer conformation at a given phi/psi using MMFF94S and QM separately.

| phi (deg) | psi (deg) | chi 1 | chi 2 | Probability (%) | ΔE (MMFF94S, kcal/mol) | ΔE (QM, kcal/mol) |
| --- | --- | --- | --- | --- | --- | --- |
| -60 | -40 | -90.05 | 88.75 | 22.27 | 0.00 | 0.00 |
|  |  | 80.02 | -164.83 | 21.97 | 0.05 | 0.27 |
|  |  | -73.62 | -151.76 | 19.66 | 0.49 | 0.29 |
|  |  | 65.56 | 64.71 | 17.23 | 1.02 | 1.06 |
|  |  | 90.11 | -126.58 | 13.14 | 2.10 | 1.90 |
|  |  | -95.61 | -30.61 | 5.72 | 5.43 | 3.57 |
| -110 | 130 | -109.80 | 164.64 | 24.58 | 0.00 | 1.88 |
|  |  | 54.60 | 64.61 | 24.49 | 0.01 | 0.26 |
|  |  | 69.22 | -169.47 | 22.07 | 0.43 | 0.00 |
|  |  | -126.69 | 41.70 | 11.15 | 3.16 | 2.60 |
|  |  | 90.58 | -86.23 | 10.23 | 3.50 | 2.76 |
|  |  | -97.61 | -97.92 | 7.48 | 4.75 | 3.79 |

Table S5. Rotamer library at a given phi/psi angle generated using AutoRotLib for N-a-Methyl-L-phenylalanine (MeF). The change in energy (ΔE) was calculated from the difference between the lowest energy rotamer conformation at a given phi/psi using MMFF94S and QM separately.

| phi (deg) | psi (deg) | chi 1 | chi 2 | Probability (%) | ΔE (MMFF94S, kcal/mol) | ΔE (QM, kcal/mol) |
| --- | --- | --- | --- | --- | --- | --- |
| -60 | -40 | -69.26 | 134.43 | 17.44 | 0.00 | 0.05 |
|  |  | -74.38 | 122.10 | 16.40 | 0.25 | 0.28 |
|  |  | -74.79 | 176.41 | 13.03 | 1.17 | 0.72 |
|  |  | -169.80 | 74.73 | 12.58 | 1.31 | 0.00 |
|  |  | -170.19 | 61.26 | 11.93 | 1.52 | 0.36 |
|  |  | 70.02 | 67.03 | 9.20 | 2.56 | 2.06 |
|  |  | 64.95 | 61.99 | 8.84 | 2.72 | 2.40 |
|  |  | -161.38 | 2.81 | 7.67 | 3.29 | 4.12 |
|  |  | 100.86 | 104.34 | 2.92 | 7.16 | 3.83 |
| -110 | 130 | -164.96 | 80.13 | 19.07 | 0.00 | 0.00 |
|  |  | -165.25 | 63.92 | 17.13 | 0.43 | 0.18 |
|  |  | -59.56 | 109.12 | 11.50 | 2.02 | 0.54 |
|  |  | -54.91 | 118.12 | 11.23 | 2.12 | 1.23 |
|  |  | -144.83 | 110.31 | 10.98 | 2.21 | 2.69 |
|  |  | -126.23 | 71.74 | 10.07 | 2.56 | 3.74 |
|  |  | 59.85 | 83.73 | 8.15 | 3.40 | 1.95 |
|  |  | 73.43 | 74.05 | 6.78 | 4.14 | 2.83 |
|  |  | 42.21 | 108.29 | 5.09 | 5.28 | 2.61 |

Table S6. Rotamer library at a given phi/psi angle generated using AutoRotlib for N-a-Methyl-L-histidine (MeH). The change in energy (ΔE) was calculated from the difference between the lowest energy rotamer conformation at a given phi/psi using MMFF94S and QM separately.

| phi (deg) | psi (deg) | chi 1 | chi 2 | Probability (%) | ΔE (MMFF94S, kcal/mol) | ΔE (QM, kcal/mol) |
| --- | --- | --- | --- | --- | --- | --- |
| -60 | -40 | -74.33 | -70.14 | 8.08 | 0.00 | 0.96 |
|  |  | -70.02 | -63.04 | 7.99 | 0.04 | 0.79 |
|  |  | 70.24 | 84.26 | 6.72 | 0.74 | 1.72 |
|  |  | 64.65 | 71.07 | 5.62 | 1.45 | 2.07 |
|  |  | 75.73 | 113.86 | 5.38 | 1.63 | 1.31 |
|  |  | -74.04 | -23.21 | 4.93 | 1.98 | 0.05 |
|  |  | -80.68 | -111.06 | 4.91 | 1.99 | 1.24 |
|  |  | -174.66 | 59.07 | 4.56 | 2.29 | 1.23 |
|  |  | -165.24 | -94.61 | 4.53 | 2.31 | 0.68 |
|  |  | -174.57 | 66.71 | 4.48 | 2.36 | 0.94 |
|  |  | -165.69 | -113.47 | 4.12 | 2.70 | 0.16 |
|  |  | 72.74 | 155.69 | 4.02 | 2.79 | 2.65 |
|  |  | -166.31 | 23.72 | 3.91 | 2.90 | 2.34 |
|  |  | -70.20 | 145.17 | 3.77 | 3.05 | 0.00 |
|  |  | -70.43 | 158.61 | 3.73 | 3.09 | 0.50 |
|  |  | -150.68 | -70.47 | 3.60 | 3.24 | 2.80 |
|  |  | -75.43 | 117.33 | 3.34 | 3.54 | 0.16 |
|  |  | 59.73 | -157.90 | 3.30 | 3.58 | 3.78 |
|  |  | -85.15 | 21.95 | 2.96 | 4.02 | 0.96 |
|  |  | 69.09 | -118.47 | 2.78 | 4.28 | 2.14 |
|  |  | -160.15 | 107.12 | 2.38 | 4.89 | 2.35 |
|  |  | -161.52 | -155.99 | 2.23 | 5.15 | 2.62 |
|  |  | 71.38 | 28.43 | 1.43 | 6.92 | 3.13 |
|  |  | 90.52 | -76.26 | 1.24 | 7.50 | 3.30 |
| -110 | 130 | -160.15 | 95.13 | 7.94 | 0.00 | 1.27 |
|  |  | -155.06 | 113.61 | 7.73 | 0.11 | 2.30 |
|  |  | -170.28 | -160.10 | 7.36 | 0.30 | 2.51 |
|  |  | -170.58 | -155.88 | 7.36 | 0.31 | 2.18 |
|  |  | -169.65 | 68.90 | 7.12 | 0.44 | 1.32 |
|  |  | -174.89 | -113.26 | 6.58 | 0.75 | 0.00 |
|  |  | -55.11 | -75.63 | 5.09 | 1.78 | 1.65 |
|  |  | -59.68 | -64.58 | 4.92 | 1.91 | 2.08 |
|  |  | -159.99 | -73.76 | 4.67 | 2.12 | 1.71 |
|  |  | -121.23 | 99.81 | 4.40 | 2.36 | 4.42 |
|  |  | -126.42 | 73.78 | 4.39 | 2.37 | 4.86 |
|  |  | -161.07 | 25.14 | 4.15 | 2.60 | 2.34 |
|  |  | -115.95 | 107.19 | 4.01 | 2.73 | 4.36 |
|  |  | -55.30 | -107.26 | 3.80 | 2.95 | 1.22 |
|  |  | 74.73 | 85.88 | 3.04 | 3.85 | 3.31 |
|  |  | -80.09 | -23.18 | 2.90 | 4.03 | 4.09 |
|  |  | 78.30 | 69.96 | 2.40 | 4.79 | 4.13 |
|  |  | 65.09 | -101.21 | 2.38 | 4.82 | 2.96 |
|  |  | 61.65 | 108.54 | 2.33 | 4.91 | 3.08 |
|  |  | 74.09 | -109.95 | 2.27 | 5.00 | 3.58 |
|  |  | -7.00 | -79.03 | 1.61 | 6.39 | 4.21 |
|  |  | -106.36 | 164.30 | 1.41 | 6.91 | 5.90 |
|  |  | 74.01 | -154.55 | 1.11 | 7.88 | 8.48 |
|  |  | 122.30 | -68.51 | 1.05 | 8.10 | 6.58 |

Table S7. Comparison of previously characterized mutations to the PUMA peptide sequence and the △ddg calculated by Rosetta.

| PUMA | Kd (nM) | △ddg | Agreement | Agreement when ddg < -1 or > 1 |
| --- | --- | --- | --- | --- |
| E132A | 0.27^#^ | 0.00 | yes | n/a |
| E136A | 0.5^#^ | 1.12 | yes | yes |
| R143A | 0.149^#^ | 0.01 | yes | n/a |
| D147A | 0.18^#^ | 0.22 | no | n/a |
| R154A | 0.28^#^ | 0.01 | yes | n/a |
| E132G | 0.34^#^ | 0.01 | yes | yes |
| E136G | 1.6^#^ | 1.03 | yes | yes |
| A139G | 0.44^#^ | 0.09 | yes | n/a |
| R143G | 0.85^#^ | 0.02 | yes | n/a |
| D147G | 1.01^#^ | 0.31 | yes | yes |
| A150G | 0.62^#^ | 0.01 | yes | n/a |
| R154G | 0.35^#^ | 0.01 | yes | n/a |
| W133F | 0.66^#^ | 0.23 | yes | n/a |
| I137A | 2.7^#^ | 3.99 | yes | yes |
| L141A | 423^#^ | 4.67 | yes | yes |
| L148A | 102^#^ | 3.52 | yes | yes |
| Y152A | 6.7^#^ | 2.62 | yes | yes |
| I137A | 19^*^ | 3.99 | yes | yes |
| I137Abu | 11^*^ | 2.52 | yes | yes |
| I137tBu | 7^*^ | -0.60 | no | n/a |
| I137Cha | 14.3^*^ | 2.81 | yes | yes |
| I137F | 17.7^*^ | 0.66 | yes | n/a |
| I137hSM | 37^*^ | 2.26 | yes | yes |
| I137DAI | 500^*^ | 4.41 | yes | yes |
| Y152Bzt | 4.5^*^ | -1.66 | no | no |
| A139tBu | 0.36^*^ | -0.78 | yes | yes |
| A144Cha | 0.23^*^ | 3.26 | no | no |
| A145G | 0.24^*^ | 0.85 | no | n/a |
| Q140Y | 0.31^*^ | -0.16 | yes | n/a |

^#^ from (Rogers et al., 2014), WT Kd reported 0.181 nM

^*^ from (Rogers et al., 2018), WT Kd reported 4 nM

Table S8. Backbone partial charge set for NCAAs.

|  | Standard alpha AA | N-cyclized AA | CA-branched or CA-cycled | N-methyl | Peptoid |
| --- | --- | --- | --- | --- | --- |
| N | -0.6046 | -0.3730 | -0.5200 | -0.3242 | -0.3242 |
| CM | n/a | n/a | n/a | -0.0999 | n/a |
| HM1/2/3 | n/a | n/a | n/a | 0.0333 | n/a |
| H | 0.3998 | 0.0257 | 0.3900 | n/a | n/a |
| CA | 0.0900 | 0.1158 | 0.1300 | 0.1968 | 0.0608 |
| HA(1) | 0.1158 | n/a | n/a | 0.1274 | 0.1317 |
| HA2 | n/a | n/a | n/a | n/a | 0.1317 |
| C | 0.6885 | 0.6885 | 0.6284 | 0.6195 | 0.6081 |
| O | -0.6885 | -0.6885 | -0.6284 | -0.6195 | -0.6081 |

Table S9. Packing test of rotameric side chains with rotamer probabilities determined different k_B_T values

| Amino Acid | Rotamer Recovery (%) | |
| --- | --- | --- |
|  | AutoRotLib, k_B_T = 1.5 | AutoRotLib, k_B_T = 4.0 |
| L-cysteine | 94.1 | 95.4 |
| L-serine | 94.0 | 96.0 |
| L-threonine | 94.0 | 96.0 |
| L-valine | 98.1 | 99.2 |
| L-leucine | 89.2 | 93.7 |
| L-isoleucine | 92.9 | 94.9 |
| L-methionine | 61.0 | 64.1 |
| L-arginine | 22.8 | 54.5 |
| L-lysine | 72.0 | 77.6 |

Table S10. Solvent exposure for residues within PUMA and CP2 were measured separately by calculating the solvent accessible surface area (SASA) of the residue in complex to its target protein and dividing by the SASA calculated for the unbound state.

| ​​PUMA Residue | Solvent Exposed (%) |  | CP2 Residue | Solvent Exposed (%) |
| --- | --- | --- | --- | --- |
| E131 | 82.1% |  | d-Tyr-1 | 100.0% |
| E132 | 100.0% |  | V2 | 40.4% |
| W133 | 57.1% |  | Y3 | 64.2% |
| A134 | 24.1% |  | N4 | 20.4% |
| R135 | 87.1% |  | T5 | 48.3% |
| E136 | 93.2% |  | R6 | 7.7% |
| I137 | 0.0% |  | S7 | 18.1% |
| G138 | 0.6% |  | G8 | 1.0% |
| A139 | 100.0% |  | W9 | 18.3% |
| Q140 | 35.1% |  | R10 | 18.7% |
| L141 | 1.0% |  | W11 | 27.1% |
| R142 | 54.3% |  | Y12 | 84.6% |
| R143 | 100.0% |  | T13 | 73.6% |
| A144 | 17.5% |  | C14 | 100.0% |
| A145 | 0.0% |  |  |  |
| D146 | 49.6% |  |  |  |
| D147 | 100.0% |  |  |  |
| L148 | 21.8% |  |  |  |
| N149 | 1.2% |  |  |  |
| A150 | 100.0% |  |  |  |
| Q151 | 100.0% |  |  |  |
| Y152 | 33.3% |  |  |  |
| E153 | 79.4% |  |  |  |
| R154 | 100.0% |  |  |  |
| R155 | 94.7% |  |  |  |
| R156 | 59.0% |  |  |  |

Table S11. Electron correlation scores for select failed cases.

| PDB ID | 4IUC | 1SVD | 3EGV | 2D7C | 3KF6 |
| --- | --- | --- | --- | --- | --- |
| residue | Phe 838 | Gln 452 | Trp 36 | Met 199 | Arg 237 |
| Full interface repacking: Δ elec correlation † | | | | | |
| Dunbrack | 0.075 | 0.082 | 0.012 | 0.000 | 0.022 |
| MakeRotLib | 0.476 | 0.446 | 0.423 | 0.414 | 0.414 |
| AutoRotLib | 0.460 | 0.451 | 0.027 | 0.032 | 0.243 |
| Single residue repacking: Δ elec correlation ‡ | | | | | |
| MakeRotLib | 0.084 | 0.022 | 0.156 | 0.423 | 0.015 |
| AutoRotLib | 0.091 | 0.003 | 0.026 | 0.032 | 0.023 |

†  Δ elec corr = electron correlation of native - electron correlation of repacked side chain. A value < 0.12 is considered successfully recovered.

‡ Reporting the Δ elec corr for the best scoring rotamer generated

**
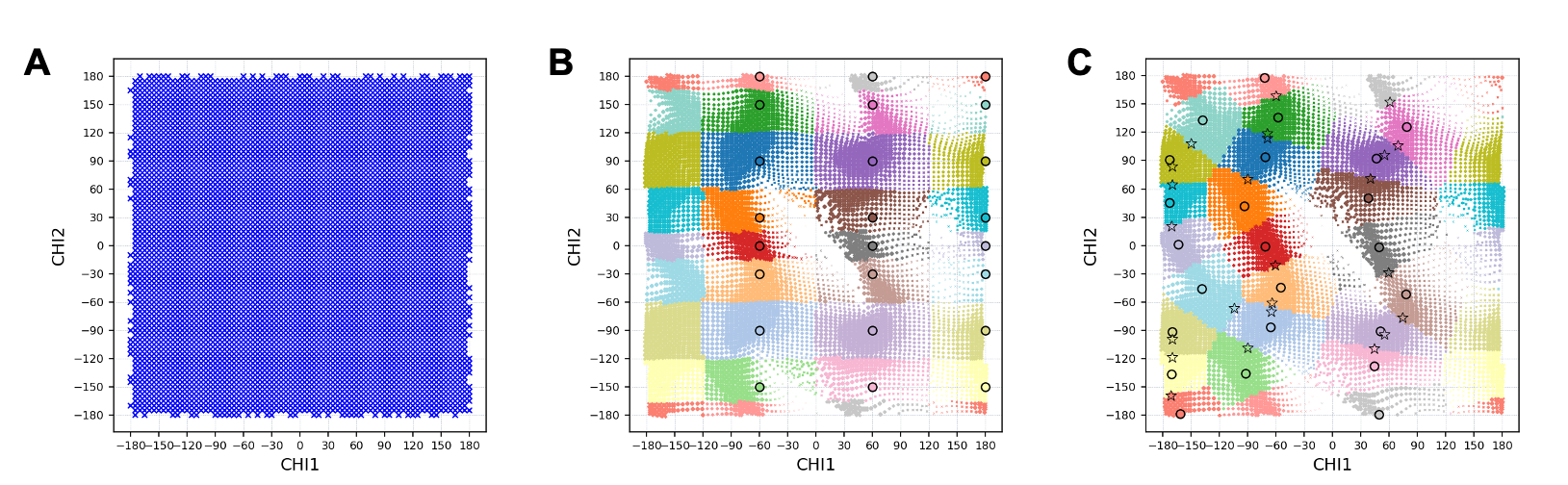
**

**Figure S1. Clustering of b-(2-Naphthyl)-L-alanine. A.** Initial side chain orientations samples for the two 𝚾 angles of the b-(2-Naphthyl)-L-alanine on a fixed backbone orientation with phi = -60° and psi = -40°. **B.** Energy optimized positions of each starting side chain orientation colored by clustering to the nearest initial cluster center (large, outlined point). **C.** Final cluster assignments of each optimized side chain orientation after two rounds of reclustering. Starred orientation represents the lowest-energy state from each cluster which is subsequently selected as a final rotamer.


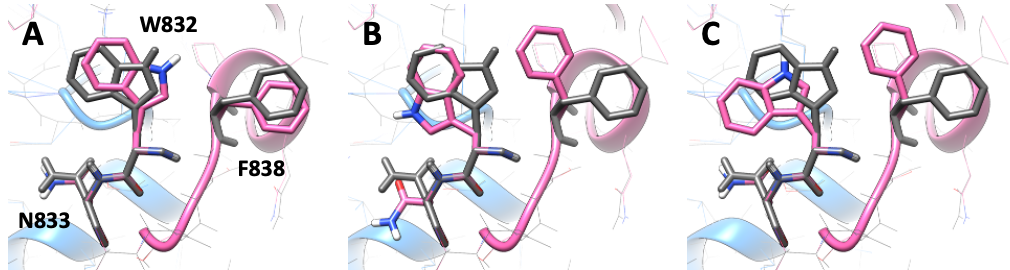


**Figure S2.** Interface rotamer packing for PDB ID: 4IUC, a O2-tolerant [NiFe] hydrogenase that is composed of a large subunit (blue) and small subunit (pink). These figures are focusing on F838 which is incorrectly packed by MRL and ARL. Neighboring residues at the interface (pink and blue chains) W832 and N833 are also highlighted. Interface packing results are shown for A.) Dunbrack rotamer libraries, B.) ARL rotamer libraries, and C.) MRL rotamer libraries. Crystallographic coordinates are shown in grey.

**Figure S3.**Fraction agreement calculated at each residue position using rotamer libraries for canonical amino acids from the Dunbrack Library represented as green squares, rotamer libraries for canonical amino acids generated from AutoRotlib are represented as black triangles and non-canonical amino acid rotamer libraries generated from AutoRotlib are represented as red triangles for A) PUMA and B) CP2.

*Analysis of Unrecovered Rotamers with MakeRotLib and AutoRotLib in CAA Interface Packing Test*

We took a closer look at some of the cases in which MakeRotLib and/or AutoRotlib failed to recover certain side chain conformations in our interface packing test and have added these details to the supplemental section. Specifically, we took a detailed look at the rotamers generated for the five positions that MRL deviated most significantly from the deposited coordinates in the PDB: Phe 838 from 4IUC; Gln 452 from 1SVD; Trp 36 from 3EGV; Met 199 from 2D7C; and Arg 237 from 3KF6. As shown in Table S1, these are all cases in which packing with the MakeRotLib-generated rotamer libraries resulted in non-recovered side chain conformations. Packing with the Dunbrack libraries resulted in recovery of all five of these positions, while using AutoRotLib-generated libraries recovered two out of five.

There are many reasons that can lead to a failed side chain recovery in a packing test. One more obvious way is that a native-like rotamer is never loaded into the packing algorithm, which makes it impossible to correctly recover. To probe this particular scenario, we ran packing tests in which only the residues in question were allowed to repack (all surrounding side chains are kept rigid with the coordinates deposited in the PDB). As shown in Table S11, MakeRotLib generates near-native rotamers for only three out of the five cases, while AutoRotLib generates near-native rotamers for all five cases. Based on this limited analysis, it seems that AutoRotLib generates more near-native rotamers than MakeRotLib.

A closer examination of the full interface packing results for Phe 838 of 4IUC, shows one way in which a residue for which a native rotamer is sampled during a packing trajectory is ultimately not selected. For both MakeRotLib and AutoRotLib, Phe 838 packs incorrectly because a neighboring residue, Trp 832 also packs incorrectly, as shown in Figure S2. This propagation of incorrectly packed side chains is often related to the score function not successfully scoring the native-like combination of rotamers as the lowest-scoring combination. This could be the result of further propagative effects of other nearby residues packing incorrectly due to a missing native rotamer or more complicated scoring issues. In this case, we also tested Trp 832 in the single-residue packing test and found that both MakeRotLib and AutoRotLib generate a native-like Trp 832 rotamer. Thus, the incorrect rotamer selection of Phe 838 for MakeRotLib and AutoRotLib is due to a different more energetically favorable state being found.

*Deep Mutational Scanning on PUMA*

The NMR ensemble of MCL-1 complexed to PUMA peptide obtained from PDB 2ROC was separated into separate PDB files that were each pre-processed with clean_pdb.py (downloaded from <https://github.com/harryjubb/pdbtools/blob/master/clean_pdb.py>). A resfile was generated to include all residues within 8 A of PUMA and all PUMA residues with the NATAA flag. The pre-processed ensemble was then minimized with FastRelax to identify a low energy model using the following command:

<path-to-Rosetta>/main/bin/relax.default.linuxccrelease -nstruct 100 -in:file:fullatom -in:file:l <input_pdb_list> -relax:respect_resfile -packing:resfile <input_resfile> -relax:ramp_constraints false -relax:script InterfaceRelax2019.txt -ex1 -ex2 -extrachi_cutoff 0 -beta -corrections::beta_nov16_cart -fa_max_dis 9.0 -overwrite

After identifying the lowest energy model the resfile was modified to indicate positional mutations with the PIKAA flag. FastDesign was then run using the following command:

<path-to-Rosetta>/main/bin/rosetta_scripts.linuxccrelease -parser:protocol <input xml> -in:file:s <input pdb> -in:file:fullatom true -ex1 -ex2 -extrachi_cutoff 0 -nstruct 200 -fa_max_dis 9.0 -overwrite -extra_res_fa <parameter file> -extra_improper_file <torsion file>

The xml template file that was used for each mutation was the following:

<ROSETTASCRIPTS>

<SCOREFXNS>

<ScoreFunction name="beta" weights="beta_nov16_cart" >

<Reweight scoretype="ref" weight="0.0" />

</ScoreFunction>

</SCOREFXNS>

<RESIDUE_SELECTORS>

<Chain name="protein" chains="1"/>

<Chain name="peptide" chains="2" />

</RESIDUE_SELECTORS>

<SIMPLE_METRICS>

<RMSDMetric name="rmsd_A” rmsd_type="rmsd_protein_bb_heavy" residue_selector="peptide" super="1" reference_name="native_pose"/>

</SIMPLE_METRICS>

<PACKER_PALETTES>

<CustomBaseTypePackerPalette name="custom_palette" additional_residue_types="<insert amino acid to be designed" />

</PACKER_PALETTES>

<TASKOPERATIONS>

<InitializeFromCommandline name="ifcl" />

<ReadResfile name="rrf" filename="<custom resfile>" />

<ExtraRotamersGeneric name="extra_chi" ex1="1" ex2="1" extrachi_cutoff="0" />

<RestrictToRepacking name="restrict" />

<RestrictToInterface name="intonly" jump="1" distance="8.0" include_all_water="0" />

</TASKOPERATIONS>

<MOVERS>

<SavePoseMover name="native" restore_pose="0" reference_name="native_pose" />

<FastDesign name="fast_design" scorefxn="beta" task_operations="ifcl,rrf,extra_chi" repeats="3" bondangle="false" bondlength="false" min_type="dfpmin_armijo_nonmonotone" ramp_down_constraints="false" cartesian="false" batch="false" packer_palette="custom_palette" >

<MoveMap name="interface">

<Jump number="1" setting="1"/>

<Chain number="1" chi="1" bb="0"/>

<Chain number="2" chi="1" bb="1"/>

</MoveMap>

</FastDesign>

<ddG name="DDG_perresidue" scorefxn="beta" task_operations="ifcl,rrf,restrict,intonly,extra_chi" per_residue_ddg="1" packer_palette="custom_palette" repack_bound="false" repack_unbound="false" jump="1" />

<RunSimpleMetrics name="run_metrics1" metrics="rmsd" prefix="m1_" />

</MOVERS>

<FILTERS>

<IRmsd name="irmsd" jump="1" threshold="2500" scorefxn="beta" />

</FILTERS>

<APPLY_TO_POSE>

</APPLY_TO_POSE>

<PROTOCOLS>

<Add mover="native" />

<Add mover="fast_design" />

<Add mover="DDG_perresidue" />

<Add filter="SHAPE" />

<Add filter="irmsd" />

<Add mover="run_metrics1"/>

</PROTOCOLS>

<OUTPUT scorefxn="beta" />

</ROSETTASCRIPTS>

*Deep Mutational Scanning on CP2*

To prepare KDM4-CP2 for deep mutational studies Chains C and E of the KDM4-CP2 crystal structure were extracted from PDB 5LY1 and renamed chains A and B, respectively. For KDM4 a missing loop (PDB numbering 162-169) was built using remodel[(Huang et al., 2011)](https://app.readcube.com/library/a676676c-458e-41b0-8105-9b32c383b188/all?uuid=3436889114627565&item_ids=a676676c-458e-41b0-8105-9b32c383b188:f83cbc21-1c57-4a95-82b3-64e06620cf97). For CP2, the atom names corresponding to the thioether linker of the macrocycle were manually renamed as described in Figure S3.


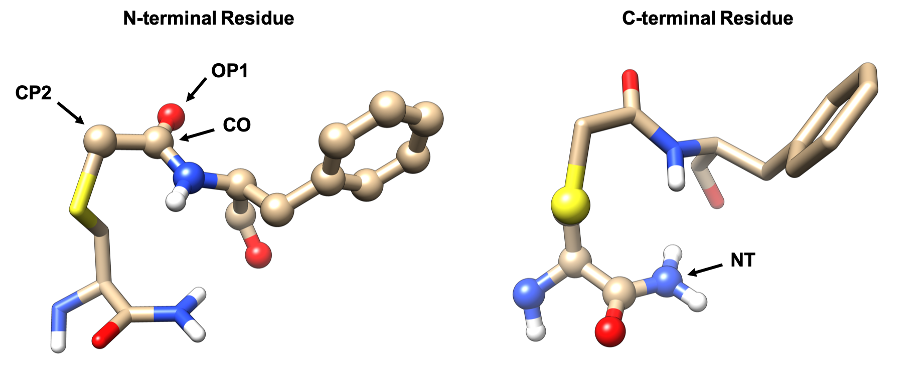


Figure S4. Atom names (CO, OP1, and CP2) of macrocycle N-acetyl linker (left) should be named accordingly as part of residue 1. C-terminal CYS residue (right) requires an 'NT' atom for the application of the CTERM_AMIDATION patch. Protons should be deleted for convenience and will be re-added by Rosetta at idealized positions.

The two modified chains of the KDM4-CP2 complex were then pre-processed using both pdb_reres.py and pdb_reatom.py (downloaded from https://github.com/haddocking/pdb-tools). Next, the complex was minimized and patched using the algorithm score_thioether_macrocycle to label the thioether bond for Rosetta algorithm as follows:

<path-to-Rosetta>/main/bin/score_thioether_macrocycle.linuxccrelease -score_thioether_macrocycle:input_file <input_pdb> -score_thioether_macrocycle:do_min -score_thioether_macrocycle:make_silent true -beta -corrections::beta_nov16

To maintain proper linker geometry, we also developed a script generate_linker_constraints.py to build a constraints file for use with Rosetta design algorithms. The minimized structure from score_thioether_macrocycle was further minimized using FastRelax. The lowest energy model produced from FastRelax was then used as an input to screen a library of canonical and NCAA residues at every residue position using FastDesign[(Maguire et al., 2020)](https://app.readcube.com/library/a676676c-458e-41b0-8105-9b32c383b188/all?uuid=22367322269000844&item_ids=a676676c-458e-41b0-8105-9b32c383b188:b15a7355-36a9-4484-a2b0-f29cb5830553). The command used to initiate FastRelax was the following:

<path-to-Rosetta>/main/bin/relax.default.linuxccrelease -nstruct 100 -in:file:fullatom -in:file:silent <input_pdb_file> -relax:respect_resfile -packing:resfile <input_resfile> -relax:ramp_constraints false -relax:script InterfaceRelax2019.txt -ex1 -ex2 -extrachi_cutoff 0 -beta -corrections::beta_nov16_cart -fa_max_dis 9.0 -constraints:cst_fa_file <constraints file> -constraints:cst_fa_weight 1.0 -overwrite

The command used for FastDesign on CP2 macrocycle was the following:

<path-to-Rosetta>/main/bin/rosetta_scripts.linuxccrelease -parser:protocol <input xml> -in::file::silent <input silent file> -in:file:silent_struct_type binary -in:file:fullatom true -ex1 -ex2 -extrachi_cutoff 0 -nstruct 200 -fa_max_dis 9.0 -overwrite -extra_res_fa <parameter file> -extra_improper_file <torsion file>

The xml template file that was used for each mutation on CP2 was the following:

<ROSETTASCRIPTS>

<SCOREFXNS>

<ScoreFunction name="beta" weights="beta_nov16_cart" >

<Reweight scoretype="atom_pair_constraint" weight="1.0"/>

<Reweight scoretype="angle_constraint" weight="1.0"/>

<Reweight scoretype="dihedral_constraint" weight="1.0"/>

<Reweight scoretype="ref" weight="0.0" />

</ScoreFunction>

</SCOREFXNS>

<RESIDUE_SELECTORS>

<Chain name="protein" chains="1"/>

<Chain name="peptide" chains="2" />

</RESIDUE_SELECTORS>

<SIMPLE_METRICS>

<RMSDMetric name="rmsd_A” rmsd_type="rmsd_protein_bb_heavy" residue_selector="peptide" super="1" reference_name="native_pose"/>

</SIMPLE_METRICS>

<PACKER_PALETTES>

<CustomBaseTypePackerPalette name="custom_palette" additional_residue_types="<insert amino acid to be designed" />

</PACKER_PALETTES>

<TASKOPERATIONS>

<InitializeFromCommandline name="ifcl" />

<ReadResfile name="rrf" filename="<custom resfile>" />

<ExtraRotamersGeneric name="extra_chi" ex1="1" ex2="1" extrachi_cutoff="0" />

<RestrictToRepacking name="restrict" />

<RestrictToInterface name="intonly" jump="1" distance="8.0" include_all_water="0" />

</TASKOPERATIONS>

<MOVERS>

<SavePoseMover name="native" restore_pose="0" reference_name="native_pose" />

<FastDesign name="fast_design" scorefxn="beta" task_operations="ifcl,rrf,extra_chi" repeats="3" bondangle="false" bondlength="false" min_type="dfpmin_armijo_nonmonotone" ramp_down_constraints="false" cartesian="false" batch="false" packer_palette="custom_palette" >

<MoveMap name="interface">

<Jump number="1" setting="1"/>

<Chain number="1" chi="1" bb="0"/>

<Chain number="2" chi="1" bb="1"/>

</MoveMap>

</FastDesign>

<ddG name="DDG_perresidue" scorefxn="beta" task_operations="ifcl,rrf,restrict,intonly,extra_chi" per_residue_ddg="1" packer_palette="custom_palette" repack_bound="false" repack_unbound="false" jump="1" />

<RunSimpleMetrics name="run_metrics1" metrics="rmsd" prefix="m1_" />

<ConstraintSetMover name="cst_mover" add_constraints="true" cst_file="csts" />

<DeclareBond name="close_cycle" res1="351" atom1="CP2" res2="364" atom2="SG" add_termini="false" rebuild_fold_tree="false" />

</MOVERS>

<FILTERS>

<IRmsd name="irmsd" jump="1" threshold="2500" scorefxn="beta" />

</FILTERS>

<APPLY_TO_POSE>

</APPLY_TO_POSE>

<PROTOCOLS>

<Add mover="native" />

<Add mover="close_cycle" />

<Add mover="cst_mover" />

<Add mover="fast_design" />

<Add mover="DDG_perresidue" />

<Add filter="SHAPE" />

<Add filter="irmsd" />

<Add mover="run_metrics1"/>

</PROTOCOLS>

<OUTPUT scorefxn="beta" />

</ROSETTASCRIPTS>

**References**

Rogers, J. M., Oleinikovas, V., Shammas, S. L., Wong, C. T., Sancho, D. D., Baker, C. M., et al. (2014). Interplay between partner and ligand facilitates the folding and binding of an intrinsically disordered protein. *Proc National Acad Sci* 111, 15420–15425. doi:10.1073/pnas.1409122111.

Rogers, J. M., Passioura, T., and Suga, H. (2018). Nonproteinogenic deep mutational scanning of linear and cyclic peptides. *Proc National Acad Sci* 115, 201809901. doi:10.1073/pnas.1809901115.
